# Supplementary material for: Induction of volatile organic compounds in chrysanthemum plants following infection by Rhizoctonia solani
Source: PLoS One. 2024 May 2;19(5):e0302541. doi: 10.1371/journal.pone.0302541 (PMC11065281; doi:10.1371/journal.pone.0302541)
Supplement: S1 Table — (DOCX) [file pone.0302541.s001.docx]

### S1 Table. *F*-statistics from two-way analysis of variance for observed VOCs

| **Source of variation** | **Cultivar** | **Collection time** | **Cultivar × collection time**  **interaction** |
| --- | --- | --- | --- |
| **Z-3-HAL** | 109.5*** | 764.56*** | 22.62*** |
| **E-2-HAL** | 60.78*** | 112.82*** | 4.28*** |
| **Z-3-HOL** | 32.77*** | 185.19*** | 4.9*** |
| **E-2-HOL** | 55.07*** | 93.73*** | 31.94*** |
| **β-PIN** | 16.49*** | 24.93*** | 2.49* |
| **β-MYR** | 24.86*** | 230.37*** | 8.97*** |
| **Z-3-HAC** | 85.94*** | 543.44*** | 11.87*** |
| **(Z)-OCI** | 58.51*** | 271.7*** | 10.01*** |
| **LIN** | 54.12*** | 361.27*** | 13.14*** |
| **BAC** | 28.91*** | 80.81*** | 5.02*** |
| **MAT** | 25.95*** | 90.53*** | 4.82*** |
| **IND** | 16.44*** | 65.34*** | 7.35*** |
| **β-CAR** | 28.44*** | 222.72*** | 7.6*** |
| **β-FAR** | 24.17*** | 433.17*** | 6.68*** |
| * P < 0.05; *** P < 0.001 | | | |
