# Supplementary material for: Induction of volatile organic compounds in chrysanthemum plants following infection by Rhizoctonia solani
Source: PLoS One. 2024 May 2;19(5):e0302541. doi: 10.1371/journal.pone.0302541 (PMC11065281; doi:10.1371/journal.pone.0302541)
Supplement: S2 Table — Mean values and standard deviations (s.d.) in columns and rows followed by the same letter do not differ significantly with two-way ANOVA and Fisher’s post-hoc test at p < 0.05. Capital letters refer to the main effects (irrespectively), small letters refer to the interaction between the two studied independent variables. (DOCX) [file pone.0302541.s002.docx]

### S2 Table. Chlorophyll *a*/*b* and chlorophylls (*a*+*b*)/carotenoids ratio in leaves of ten chrysanthemum cultivars infested with *Rhizoctonia solani* on leaves and on the surface of growth substrate. Mean values and standard deviations (s.d.) in columns and rows followed by the same letter do not differ significantly with two-way ANOVA and Fisher’s post-hoc test at *p* < 0.05. Capital letters refer to the main effects (irrespectively), small letters refer to the interaction between the two studied independent variables.

| **Treatment** | |  | **Cultivars** | | | | | | | | | | | |
| --- | --- | --- | --- | --- | --- | --- | --- | --- | --- | --- | --- | --- | --- | --- |
|  |  |  | **Ania** | | **Beata** | **Brda** | **Kasia** | **Lidka** | **Luczniczka** | **Malgosia** | **Polka** | **Wda** | **Zofia** | **Mean** |
|  |  | | | **Chlorophyll *a*/*b*** | | | | | | | | | | |
| **control** | | Mean  s.d. | 2.44cd 0.94 | | 3.42 a  1.03 | 2.45 cd  0.12 | 2.16 d  0.80 | 3.03 a-c  0.20 | 3.31 ab  0.74 | 2.66 b-d  0.58 | 3.05 a-c  0.70 | 2.63 b-d  0.37 | 3.04 a-c  0.57 | **2.82 A** |
| **leaves infestation** | | Mean  s.d. | 2.33 cd  0.26 | | 2.17 d  0.16 | 2.05 d  0.18 | 2.14 d  0.32 | 2.05 d  0.30 | 2.26 d  0.16 | 2.18 d  0.08 | 2.27 d  0.06 | 2.23 d  0.06 | 2.19 d  0.20 | **2.19 C** |
| **soil infestation** | | Mean  s.d. | 2.41 cd  0.48 | | 2.33 cd  0.24 | 2.56 cd  0.64 | 2.39 cd  0.16 | 2.54 cd  0.46 | 2.42 cd  0.36 | 2.58 b-d  0.18 | 2.48 cd  0.32 | 2.56 cd  0.43 | 2.13 d  0.17 | **2.44 B** |
| **Mean** | |  | **2.39 AB** | | **2.64 AB** | **2.35 AB** | **2.23 B** | **2.54 AB** | **2.66 A** | **2.47 AB** | **2.60 AB** | **2.47 AB** | **2.45 AB** |  |
|  | **Chlorophylls (a+*b*)/carotenoids** | | | | | | | | | | | | | |
| **control** | | Mean  s.d. | 7.21 a  1.52 | | 5.07 i  1.02 | 5.99 b-i  0.07 | 6.48 a-f  1.07 | 5.22 hi  0.25 | 5.41 g-i  0.44 | 5.56 e-i  1.40 | 5.31 g-i  0.67 | 5.85 c-i  0.59 | 5.48 g-i  0.18 | **5.76 B** |
| **leaves infestation** | | Mean  s.d. | 6.17 b-h  0.18 | | 5.87 c-i  0.48 | 6.52 a-e  0.65 | 6.47 a-f  0.40 | 6.57 a-d  0.35 | 6.53 a-e  0.37 | 5.95 b-i  0.69 | 6.14 b-h  0.26 | 6.83 a-c  0.07 | 6.28 a-g  0.08 | **6.33 A** |
| **soil infestation** | | Mean  s.d. | 6.15 b-h  0.56 | | 6.07 b-h  0.55 | 5.79 d-i  0.28 | 5.71 d-i  0.36 | 5.71 d-i  0.48 | 6.29 a-g  0.28 | 5.51 f-i  0.21 | 5.66 d-i  0.39 | 5.90 c-i  0.25 | 6.92 ab  0.63 | **5.97 B** |
| **Mean** | |  | **6.51 A** | | **5.67 B** | **6.10 AB** | **6.22 AB** | **5.83 B** | **6.08 AB** | **5.67 B** | **5.70 B** | **6.19 AB** | **6.22 AB** |  |
