# Supplementary material for: Induction of volatile organic compounds in chrysanthemum plants following infection by Rhizoctonia solani
Source: PLoS One. 2024 May 2;19(5):e0302541. doi: 10.1371/journal.pone.0302541 (PMC11065281; doi:10.1371/journal.pone.0302541)
Supplement: S3 Table — (DOCX) [file pone.0302541.s003.docx]

### S3 Table. *F*-statistics from two-way analysis of variance for observed metabolites content in leaves

| **Source of variation** | **Cultivar** | ***Rhizoctonia solani* treatment** | **Cultivar × *Rhizoctonia solani* treatment** |
| --- | --- | --- | --- |
| **Chlorophyll *a*** | 6.09*** | 2.37*** | 1.44*** |
| **Chlorophyll *b*** | 2.26* | 3.44* | 1.09* |
| **Chlorophylls (*a* + *b*)** | 4.46*** | 1.13*** | 1.26*** |
| **Carotenoids** | 7.99*** | 4.45*** | 1.36*** |
| **Phenolic compounds** | 3.01*** | 14.72*** | 2.60*** |
| **Chlorophyll *a*/*b*** | 0.78*** | 14.50*** | 1.02*** |
| **Chlorophylls (a+*b*)/carotenoids** | 2.03* | 6.93* | 1.83* |
| * *p* < 0.05; *** *p* < 0.001 | | | |
