# Supplementary material for: Induction of volatile organic compounds in chrysanthemum plants following infection by Rhizoctonia solani
Source: PLoS One. 2024 May 2;19(5):e0302541. doi: 10.1371/journal.pone.0302541 (PMC11065281; doi:10.1371/journal.pone.0302541)
Supplement: S10 Fig — (DOCX) [file pone.0302541.s013.docx]

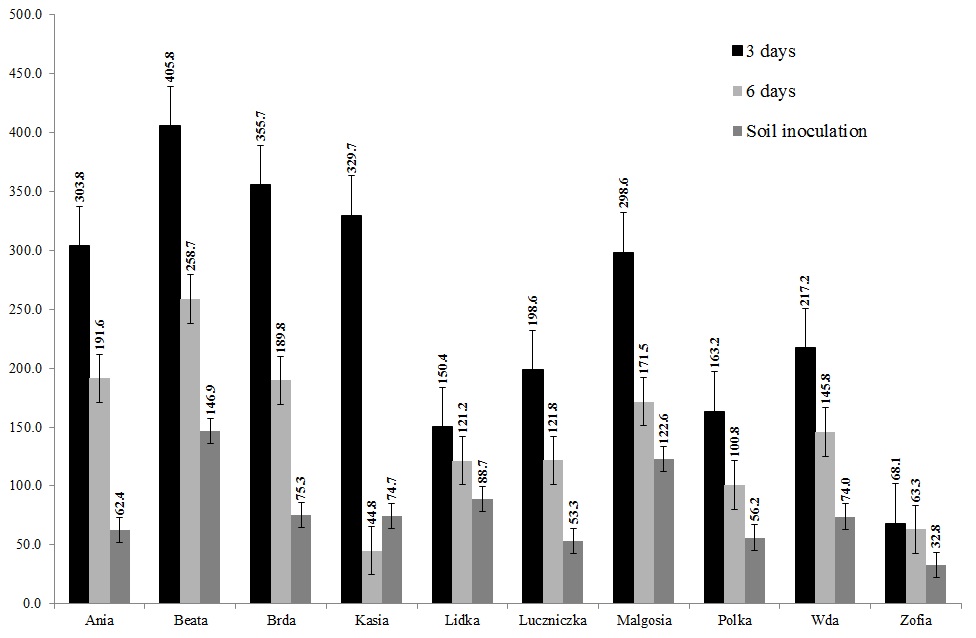


### S10 Fig. Mean values [ng hr^-1^] for LIN emission by *Chrysanthemum* × *morifolium* cultivars following the infestation of *Rhizoctonia solani,* collected on days 3 and 6 post-foliar application, and on day 42 after soil-inoculation.
