# Supplementary material for: Induction of volatile organic compounds in chrysanthemum plants following infection by Rhizoctonia solani
Source: PLoS One. 2024 May 2;19(5):e0302541. doi: 10.1371/journal.pone.0302541 (PMC11065281; doi:10.1371/journal.pone.0302541)
Supplement: Attachment — Submitted filename: Response to Reviewers-2024-02-15 CAM.docx [file pone.0302541.s019.docx]

**Dear Reviewers,**

**We appreciate the opportunity to respond to the points raised in our recent publication, titled “Induction of volatile compounds in chrysanthemum plants following infection by Rhizoctonia”. After thorough review and analysis, we find the reviews as very helpful and constructive. We have addressed all the issues raised by the Reviewers. We hope that our answers will clarify all of them. Our answers, comments and corrections are listed below the Reviewers queries, typed in red.**

Reviewer #1: This comprehensive study investigates the impact of Rhizoctonia solani infestation on ten cultivars of chrysanthemum, shedding light on both volatile organic compound (VOC) emissions and biochemical composition of plants. Through meticulous analysis using gas chromatography-mass spectrometry, the research reveals cultivar-specific variations in VOC emissions, with some displaying a broader spectrum compared to others. The investigation underscores the intricate interplay between cultivar, collection time, and Rhizoctonia infection, elucidating significant effects on VOCs. Notably, the study unveils alterations in biochemical composition induced by the infection, with a noteworthy decrease in carotenoid content and an increase in phenolics, offering valuable insights for disease management strategies and the development of resistant chrysanthemum cultivars. The manuscript is interesting and valuable, its novelty is clear. I only have some minor technical suggestions regarding the text.

Dear Reviewer #1, Thank you for your evaluation and supportive comments. We did our best to address them properly. Please, find our answers typed in red.

- Please provide the initials of the authors when mentioning the species for the first time.

It has been done.
- Some older references in the Introduction can be updated.

In the Introduction the following references (by numbers) have been updated: 10, 19, 22, 27 and 28
- Some names of the cultivars are inconsistent throughout the text, e.g. Łuczniczka and Luczniczka.

We have changed the typing of Łuczniczka to Luczniczka. Also, we have retyped Małgosia with Malgosia, following your suggestion and to keep consistency.
- According to the Abstract, the plants were grown in controlled conditions in the glasshouse, yet there is no information on the light conditions.

This information has been added to Material and methods. Also, in the Abstract, we added word “semi-” to “controlled” conditions, since the light provided to plants was from natural source.
- Avoid repetitions (see page 6).

It has been corrected.
- Sometimes in the text, you give detailed information about the producers of chemicals and equipment and sometimes only brief.

It has been corrected. All the manufacturers’ data have been adjusted to the name of the company and the country of production.
- Sometimes you use the abbreviation ml and sometimes mL (I suggest the latter one).

It has been corrected.
- Some parts of the text are unclear (see the corrected manuscript).

Thank you for all of your valuable comments in the manuscript, we have corrected all of them according to your suggestions. Thank you for your patience and time.
- Sometimes you are using a serial comma, and sometimes not.

It has been corrected, serial commas have been deleted.

- I suggest preparing a separate Conclusions chapter. What are the suggestions for future research?

The separate Conclusion chapter which presents also some future directions prospects has been added to the manuscript.
- In the Reference list, Latin names should be written in italics.

It has been corrected
- Grammar and punctuation require minor corrections.

It has been corrected
For more specific comments, please see the corrected manuscript.

We have followed all your suggestions.

Thank you for your time and effort, providing us with detailed, as well as essential comments, which have improved the quality of the manuscript.

Reviewer #2: The manuscript is about the Induction of volatile organic compounds in chrysanthemum plants following *Rhizoctonia solani* infection. The manuscript contain important information for the scientific community. I have following suggestions to improve this.

Dear Reviewer#2, Thank you for your supportive comments. Please, find our answers typed in red below.
Title: Change to “chrysanthemum plants” instead of ”chrysanthemums plants” and add “solani” after Rhizoctonia as the study is on a specific bacterial infection.

It has been corrected.
Abstract;
The aim and the significance/application of the study was not well justified.

We have developed the aim and significance of our study in the Abstract, according to your suggestion.

Introduction
Line 74- Please give some examples

The examples of certain GLVs action has been added to the sentence with new references (11, 12 and 13)

**].**
Materials and methods
Line 134- What are the greenhouse conditions?

We have added information concerning light and photoperiod conditions during the cultivation and infestation period. The temperature and humidity information had already been indicated in lines 144-146 of original manuscript.
Line 145- Do you mean (Fig.1A)? Please be consistent.

Thank you, it has been adjusted to keep consistency.
Line 183- What is super-Q absorbent?

Volatile Collection Traps (Super-Q), are passive chemical filters designed for the collection of extremely low-level (ppm-ppb) volatile organic compounds (VOC’s) from nalophan enclosed plants. This information has been added into the manuscript.
Line 204- Manufacturer of GC-MS?

The manufacturer of GC-MS was (Perkin Elmer, USA), it was indicated in line 208 of the original manuscript.
Results:
Line 290 – “figures” and Line 299 “Figs”. Please be consistent.

It has been corrected and adjusted in other places of manuscript.
Line 338- What trait?

It has been corrected. The sentence was improved with: “The mean chlorophyll a content in different cultivars regardless the *Rhizoctonia* treatments…”
Line 343- Please include the data not shown in supplementary materials to confirm significance.

The supplementary tables S2 and S3 has been included into Supplementary file.
